# Co-Crystal Structures of Inhibitors with MRCKβ, a Key Regulator of Tumor Cell Invasion

**DOI:** 10.1371/journal.pone.0024825

**Published:** 2011-09-20

**Authors:** Timo Heikkila, Edward Wheatley, Diane Crighton, Ewald Schroder, Alexandra Boakes, Sarah J. Kaye, Mokdad Mezna, Leon Pang, Mathew Rushbrooke, Andrew Turnbull, Michael F. Olson

**Affiliations:** 1 Cancer Research Technology Discovery Laboratories, Wolfson Institute for Biomedical Research, London, United Kingdom; 2 Beatson Institute for Cancer Research, Glasgow, United Kingdom; Bauer Research Foundation, United States of America

## Abstract

MRCKα and MRCKβ (myotonic dystrophy kinase-related Cdc42-binding kinases) belong to a subfamily of Rho GTPase activated serine/threonine kinases within the AGC-family that regulate the actomyosin cytoskeleton. Reflecting their roles in myosin light chain (MLC) phosphorylation, MRCKα and MRCKβ influence cell shape and motility. We report further evidence for MRCKα and MRCKβ contributions to the invasion of cancer cells in 3-dimensional matrix invasion assays. In particular, our results indicate that the combined inhibition of MRCKα and MRCKβ together with inhibition of ROCK kinases results in significantly greater effects on reducing cancer cell invasion than blocking either MRCK or ROCK kinases alone. To probe the kinase ligand pocket, we screened 159 kinase inhibitors in an *in vitro* MRCKβ kinase assay and found 11 compounds that inhibited enzyme activity >80% at 3 µM. Further analysis of three hits, Y-27632, Fasudil and TPCA-1, revealed low micromolar IC_50_ values for MRCKα and MRCKβ. We also describe the crystal structure of MRCKβ in complex with inhibitors Fasudil and TPCA-1 bound to the active site of the kinase. These high-resolution structures reveal a highly conserved AGC kinase fold in a typical dimeric arrangement. The kinase domain is in an active conformation with a fully-ordered and correctly positioned αC helix and catalytic residues in a conformation competent for catalysis. Together, these results provide further validation for MRCK involvement in regulation of cancer cell invasion and present a valuable starting point for future structure-based drug discovery efforts.

## Introduction

Tumor cell metastasis is a multi-step process driven by dynamic reorganization of the actomyosin cytoskeleton and remodeling of the extracellular matrix that allows cells to cross tissue boundaries and spread via blood and lymphatic vessels to distal regions of the body [1]. Members of the Rho GTPase family are key regulators of the actomyosin cytoskeleton required for the processes associated with invasion and metastasis [2]. The bundling and contraction of actin-myosin fibers provides the force required for cell motility and invasion [1]. On this basis, downstream effector proteins such as the Rho-regulated ROCK1 and ROCK2 protein kinases that directly impact upon actomyosin contractility have emerged as attractive potential targets for anti-metastatic therapeutics [3], [4]. ROCK inhibitors have been shown to reduce the invasive ability of tumor cells *in vitro* and to prevent the *in vivo* dissemination of tumor cells including melanoma, fibrosarcoma, liver, breast, lung and prostate cancer [5]–[11].

Recent research has shown that there are multiple modes of individual tumor cell invasion with differing sensitivities to ROCK inhibition [12]–[14]. Cells that migrate through 3-dimensional (3-D) extracellular matrix (ECM) with a rounded morphology (also known as amoeboid invasion) are more dependent upon ROCK activity, whereas cells that invade using elongated actin-rich protrusions (also called mesenchymal invasion) are relatively insensitive to ROCK inhibition [15]–[18]. However, both invasion modes are dependent upon the contractile force generated by myosin ATPase activity [17], indicating that regulators of actomyosin function in addition to ROCK are involved.

Cdc42 is a member of the Rho GTPase protein family that plays key roles in actomyosin cytoskeletal organization and cell migration through effector proteins including the myotonic dystrophy kinase-related Cdc42-binding kinases α and β (MRCKα and MRCKβ) [19]. Both ROCK and MRCK belong to the AGC kinase family, and MRCK can be further classified into the myotonic dystrophy protein kinase (DMPK) subfamily. MRCKα and MRCKβ are 190 kDa multi-domain proteins expressed in a wide range of tissues, with ∼80% sequence identity across their kinase domains. ROCK and MRCK kinases share ∼45–50% sequence identity homology over the N-terminal kinase domains, which is reflected in their shared abilities to phosphorylate a similar set of substrates (such as the myosin binding subunit (MYPT1) of the myosin light chain (MLC) phosphatase complex [17], [20]–[22]). However, the C-terminal regulatory regions of ROCK and MRCK are distinctly different. Importantly, it has been observed that actomyosin contractility required for the invasion of cells with elongated mesenchymal morphology is dependent on Cdc42-MRCK signaling [17]. In such cells, which were largely resistant to ROCK inhibition alone, siRNA-mediated knockdown of MRCK had some effect on inhibiting invasion while the combination of MRCK knockdown along with ROCK inhibition more effectively inhibited invasion and caused cells to adopt a spherical, non-blebbing morphology. These data indicate that during elongated mesenchymal invasion, ROCK and MRCK regulate independent and co-operative pathways that collaborate in a non-compensatory manner. Given that there appears to be considerable plasticity in the abilities of tumor cells to interchange between elongated and rounded modes of tumor cell invasion in response to varying environmental circumstances [12]–[14], one potential anti-invasion strategy would be to simultaneously target ROCK and MRCK activity in order to inhibit multiple invasion modes and to counteract tumor cell adaptability.

Further data supporting the strategy of simultaneous ROCK and MRCK inhibition comes from organotypic cell culture systems used to examine ECM invasion by co-cultures of squamous cell carcinoma (SCC) and cancer-associated stromal fibroblasts (CAF) [23]. SCC cells form an epidermal-like layer when grown on a three-dimensional collagen matrix, within which embedded CAFs are able to create paths in the collagen layer that enable SCCs to leave the epidermal layer and invade. The ability of tumor derived fibroblasts to generate paths is dependent on ROCK activity to remodel the matrix, while the ability of the SCCs to move through the CAF-generated paths can be blocked by MRCK knockdown [23]. The critical contribution of MRCK in collective invasion apparently is to provide actomyosin contractility around the periphery that helps to maintain cohesion of the cell collective [23], [24]. These data indicate that as well as blocking the ability of tumor cells to alternate between invasion modes, blocking MRCK and ROCK together would target different processes that co-operate to promote tumor cell invasion.

In this study we have confirmed that the greatest inhibition of 3-D ECM invasion by MDA MB 231 breast cancer cells occurs with the combined inhibition of MRCK and ROCK. To examine the structural basis of MRCK activity and to explore the potential for developing specific inhibitors, we screened a collection of kinase inhibitors and identified several that inhibited MRCK with low micromolar IC_50_ values. Furthermore, we determined the structure of MRCKβ in complex with two ATP-competitive inhibitors, namely Fasudil and TPCA-1. These results and crystal structures provide valuable starting points for the development of compounds that could potentially be used as anti-metastatic therapeutics.

## Results

### Combined MRCK and ROCK inhibition reduces 3-dimensional matrix invasion

**Figure 1:**
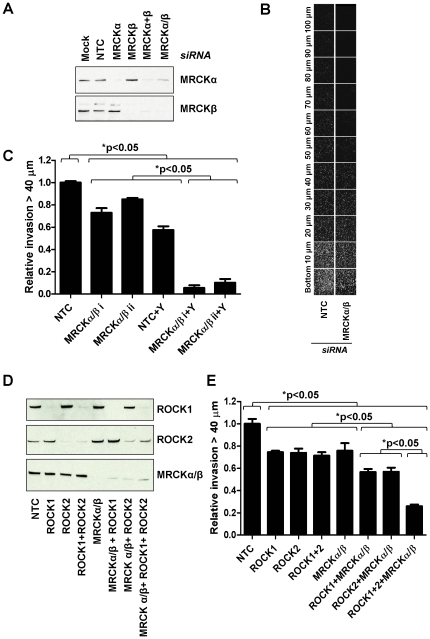
Inhibition of 3-D matrigel invasion by MDA MB 231 cells following MRCK and ROCK inhibition. (**A**) Knock-down of MRCKα and MRCKβ individually or in combination. Double knockdown was achieved either by combining separate siRNAs (MRCKα+β) or single siRNA duplexes that target both kinases (MRCKα/β). NTC = non-targeting control. (**B**) Optical slices were obtained every 10 µm by confocal imaging. (**C**) Invasion 40 µm above the transwell filter surface was normalized to non-targeting control (NTC) siRNA transfected cells. Significant differences between groups of columns indicated. (Average ± SEM, n = 3). MRCKα/β knockdown or ROCK inhibition with Y-27632 (Y) significantly decreased invasion, with the combination of MRCKα/β knockdown and ROCK inhibition resulting in significantly more inhibition. (**D**) Effectiveness and specificity of ROCK1, ROCK2 and MRCKαβ knockdowns. (**E**) The combination of MRCKα/β with ROCK1 and ROCK2 knockdown individually or in combinations were tested for their effects on 3-D matrigel invasion. ROCK1, ROCK2 or ROCK1+ROCK2 combination were able to significantly inhibit invasion. However, additional MRCKα/β knockdown significantly increased the inhibition of invasion in each instance. (Average ± SEM, n = 3).

The contribution of MRCK to tumor cell invasion was examined by knocking down both MRCKα and MRCKβ in MB 231 breast cancer cells and determining the effects in a 3-dimensional inverse matrigel invasion assay [25]. The combined MRCKα plus MRCKβ knockdown could be achieved either with two siRNA duplexes targeting each mRNA transcript (MRCKα+β) or with a single siRNA duplex (MRCKα/β) that targets both (Figure 1A). Following plating on the underside of Transwell inserts containing a thick layer of matrigel and allowing 5 days for invasion through the porous filter and into the matrigel, the extent of MDA MB 231 cell invasion was determined by fixing and staining cells with propidium iodide, followed by confocal microscopic optical sectioning at 10 µm intervals (Figure 1B). The combined knockdown of MRCKα/β with two independent doubly-targeting siRNA duplexes significantly reduced invasion relative to non-targeted control (NTC) siRNA transfected cells (Figure 1C). Treatment of NTC transfected cells with ROCK inhibitor Y-27632 also significantly reduced invasion, while the combination of MRCKα/β knockdown plus Y-27632 treatment was significantly more effective than either MRCKα/β knockdown or Y-27632 treatment alone (Figure 1C). Given the potential for off-target effects of Y-27632, particularly on highly homologous kinases such as MRCK, we knocked down ROCK 1 and/or ROCK2 to corroborate the effects of ROCK inhibition (Figure 1D). The individual knockdowns of ROCK1 or ROCK2, as well as the combined knockdown of ROCK1+ROCK2 or MRCKα/β, were sufficient to significantly inhibit invasion above 40 µm (Figure 1E). When MRCKα/β knockdown was combined with either ROCK1 or ROCK2 knockdown the effect was significantly greater than for any of these conditions alone (Figure 1E). The complete combination of MRCKα/β with ROCK1+ROCK2 knockdown was most effective of all, being significantly more inhibitory than any of the other combinations (Figure 1E). These data support the conclusion that the most effective method to reduce tumor cell invasion is through the combined inhibition of ROCK and MRCK signaling.

### Kinase inhibitor screen

**Figure 2:**
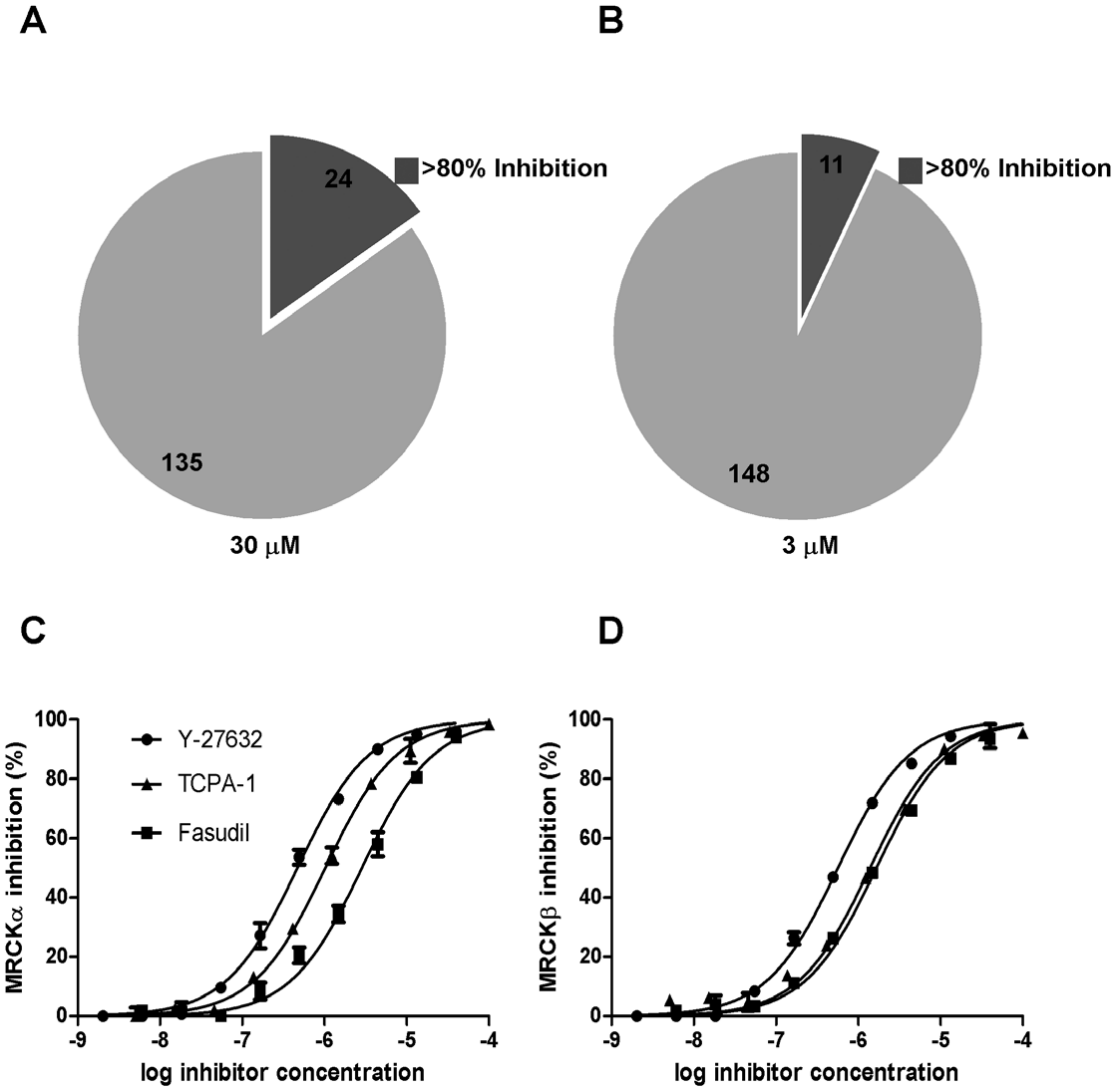
Inhibition of MRCK activity by kinase inhibitors. A collection of 159 kinase inhibitors were tested for their ability to inhibit MRCKβ activity in vitro at (**A**) 30 µM and (**B**) 3 µM. Pie charts represent the proportion inhibiting >80% at each concentration. Inhibition of (**C**) MRCKα or (**D**) MRCKβ activity by Y-27632, TPCA-1 and Fasudil. Both kinases were inhibited by these compounds, although some differences in sensitivity were apparent.

In order to identify tool compounds and to begin building structure-activity relationship information on MRCK inhibitors, we screened 159 kinase inhibitors at 30 µM and 3 µM in MRCKβ *in vitro* kinase assays at the enzyme K_m_ for ATP. We found that 15% (24/159) of the inhibitors resulted in >80% inhibition at 30 µM (Figure 2A) and 7% (11/159) inhibited >80% at 3 µM (Figure 2B and Table 1). In some instances, inhibition at 30 µM was lower than at 3 µM, which was likely due to inhibitors partially coming out of solution at higher concentration. These experiments revealed that some ROCK inhibitory compounds also inhibited MRCKβ (*e.g.* H-89 99.1% inhibition at 30 µM and 79.3% inhibition at 3 µM; Fasudil 95.4% inhibition at 30 µM and 63.6% at 3 µM; Y-27632 96.6% inhibition at 30 µM and 83.5% at 3 µM), consistent with the high homology between the MRCK and ROCK kinase domains. Interestingly, the reportedly IKK2-selective inhibitor TPCA-1 [26] was also reasonably effective at inhibiting MRCKβ (88.9% inhibition at 30 µM and 50.1% at 3 µM). To more comprehensively characterize the effects of selected inhibitors, 10 point dose-response analysis over 5 log_10_ drug concentrations were carried out for Y-27632, TPCA-1 and Fasudil for MRCKα and MRCKβ. These assays revealed comparable rank orders of potencies with Y-27632>TPCA-1>Fasudil for both MRCKα and MRCKβ (Figure 2C and 2D, Table 2). The ability of Fasudil to inhibit MRCK activity suggests that this inhibitor may not be as strictly selective for ROCK kinases as had previously been indicated [27], in agreement with the reported incomplete selectivity of this compound [28]. The observation that Fasudil did not effectively inhibit MRCK activity in Amano *et al.* [27] likely results from differences in assay conditions (*e.g.* >140 times higher ATP concentration).

**Table 1:** Inhibitors of MRCKβ *in vitro*.

Inhibitor	Inhibition at 3 µM (%)	Inhibition at 30 µM (%)
Alsterpaullone	90.7	78.4
Alsterpaullone, 2-Cyanoethyl	98.4	94.8
Bisindolylmaleimide I	87.9	ND
Cdk1 Inhibitor, CGP74514A	87.2	100
Cdk1/2 Inhibitor III	100	100
Gö 6983	88.6	65.1
K-252a, Nocardiopsis sp.	87.3	98.4
PKR Inhibitor	80.1	100.5
Ro-32-0432	85.2	68.2
Staurosporine	98.6	100

**Table 2:** IC_50_ values obtained for Fasudil, TPCA-1 and Y-27632 *in vitro*.

Inhibitor	IC_50_ (µM)	
	MRCKα	MRCKβ
Fasudil	3.13±0.41	1.92±0.36
TPCA-1	1.04±0.06	1.69±0.13
Y-27632	0.37±0.05	0.47±0.03

### MRCKβ Structure

#### Active conformation despite lack of phosphorylation

**Figure 3:**
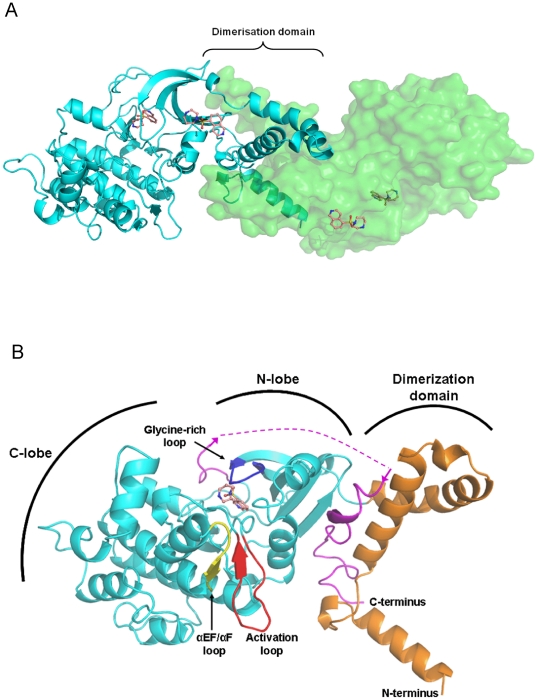
Structure of dimeric MRCKβ. (**A**) Overall structure of the dimeric MRCKβ shows the interactions of the two monomers at the dimerization domain. The four Fasudil molecules observed per asymmetric unit are also shown, two bound to the surface of the protein (central) and one bound to each of the ATP-binding sites (lateral). (**B**) A close-up of one monomer reveals a typical two-lobed kinase structure, with both the N-terminus (orange) and the C-terminus (purple; disorganized loop shown as a dashed line) forming the dimerization domain. The glycine-rich loop (blue) and activation loop (red) are fully ordered, and the αEF/αF-loop is also indicated (yellow). Fasudil is shown bound in the ATP-binding site.

**Figure 4:**
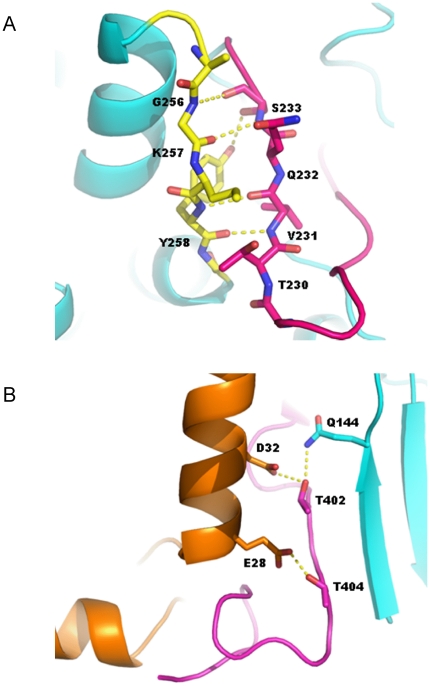
Detailed views of intramolecular interations. (**A**) Interactions involved in the small antiparallel β-sheet formed from a part of the activation loop (red) and a part of the αEF/αF loop (yellow). (**B**) Hydrogen bonding interactions involved in the stabilization of the hydrophobic motif within the dimerization domain.

The overall structure bears great resemblance to the closest AGC kinase homologues, DMPK and ROCK1/2. The quaternary structure of the MRCKβ kinase domain is dimeric, with both the N- and C-termini involved in the dimerization interface (Figure 3A and 3B). In both monomers, the activation loop is well-ordered and in a conformation that does not impede access to the nucleotide or substrate binding sites, thus resembling an active kinase conformation. As in the ROCK and DMPK kinase domain structures [29]–[31], an extension in the length of the activation loop relative to that in PKA [32] allows a small antiparallel β-sheet to be produced from a part of the activation loop and a part of the αEF/αF loop (Figure 3B and 4A). The conservation of these structural features between MRCK and AGC kinase homologues implies that the interactions between the activation loop and the αEF/αF loop are important in stabilizing the activation loop in the open conformation. The active conformation of the activation segment is additionally stabilized by a hydrogen bond formed by the catalytic loop (HRD) arginine (R199) with the main chain carbonyl of S221 located C-terminally to the DFG motif. The catalytic aspartate of the HRD motif (D200) is in a suitable position for catalysis. The helix αC in close proximity to the active site is fully ordered in the MRCK structure, and the salt bridge between αC and β3 that is an indicator of an active kinase conformation is present (residues E124 and K105).

It has been previously reported that MRCK requires dimerization and trans-autophosphorylation for activation, with mutational analysis suggesting important regulatory roles of phosphorylation sites at S234, T240, and T403 on MRCKα [33] (S233, T239 and T402 in MRCKβ respectively). As with the structures of other related AGC kinases [29], [30], no density consistent with phosphorylation was observed at these conserved residues in the MRCKβ structure. Furthermore, other AGC kinases, such as DMPK and ROCK, have been found to have enzymatic activity despite the lack of phosphorylation.

#### Conserved dimerization domain

Size-exclusion chromatography indicated that MRCKβ elutes as a dimer (data not shown). The crystallographic asymmetric unit contains two molecules of the MRCKβ kinase domain, locked in a conserved intermolecular interaction that represents the biological dimer interface. As in the structures of the related AGC kinases, the regions of MRCKβ involved in the dimer interface are the four N-terminal helices as well as the C-terminus of the kinase domain (Figure 3A and 4B).

The buried accessible surface area in the MRCKβ dimer is 2550 Å^2^, while those of ROCK1 and DMPK are 2100 Å^2^ and 1650 Å^2^ respectively (calculated using the MSD-PISA server [34] (http://www.ebi.ac.uk/msd-srv/prot_int/pistart.html). The difference between MRCKβ and DMPK can be explained by the extended α1-helix seen in the former, spanning 14 residues in comparison to only 6 residues in DMPK. The differences between ROCK1 and MRCKβ are more subtle, and the length of the α1-helix is very similar in both proteins. The C-terminus of MRCKβ can be traced further in electron density than that of ROCK1, thus contributing to the increased buried surface at the interface (Figure 3A).

#### Conserved binding of the C-terminal tail to the N-terminal lobe - the hydrophobic motif

Dimerization is facilitated through the binding of the C-terminal tail of the kinase domain to a groove in the N-terminal lobe (Figure 3A and 4B). This arrangement is a general feature of AGC kinases, where a groove is created in the N-terminal lobe by insertion of a small α-helix, which causes a separation of helix αC and strand β4. The C-terminus of the protein wraps around the N-terminal lobe to allow the binding of the hydrophobic motif on the C-terminal tail between αC and β4. This binding has occurred despite the lack of phosphorylation at the hydrophobic motif, with two pairs of polar residues in that are in hydrogen bonding distance from each others, namely E28-T404 and D32-T402 (Figure 4B). This probably represents a phosphorylation-independent stabilization mechanism for the hydrophobic motif in this family, as previously suggested for DMPK [28].

#### Binding of inhibitors to the active site of MRCKβ

**Figure 5:**
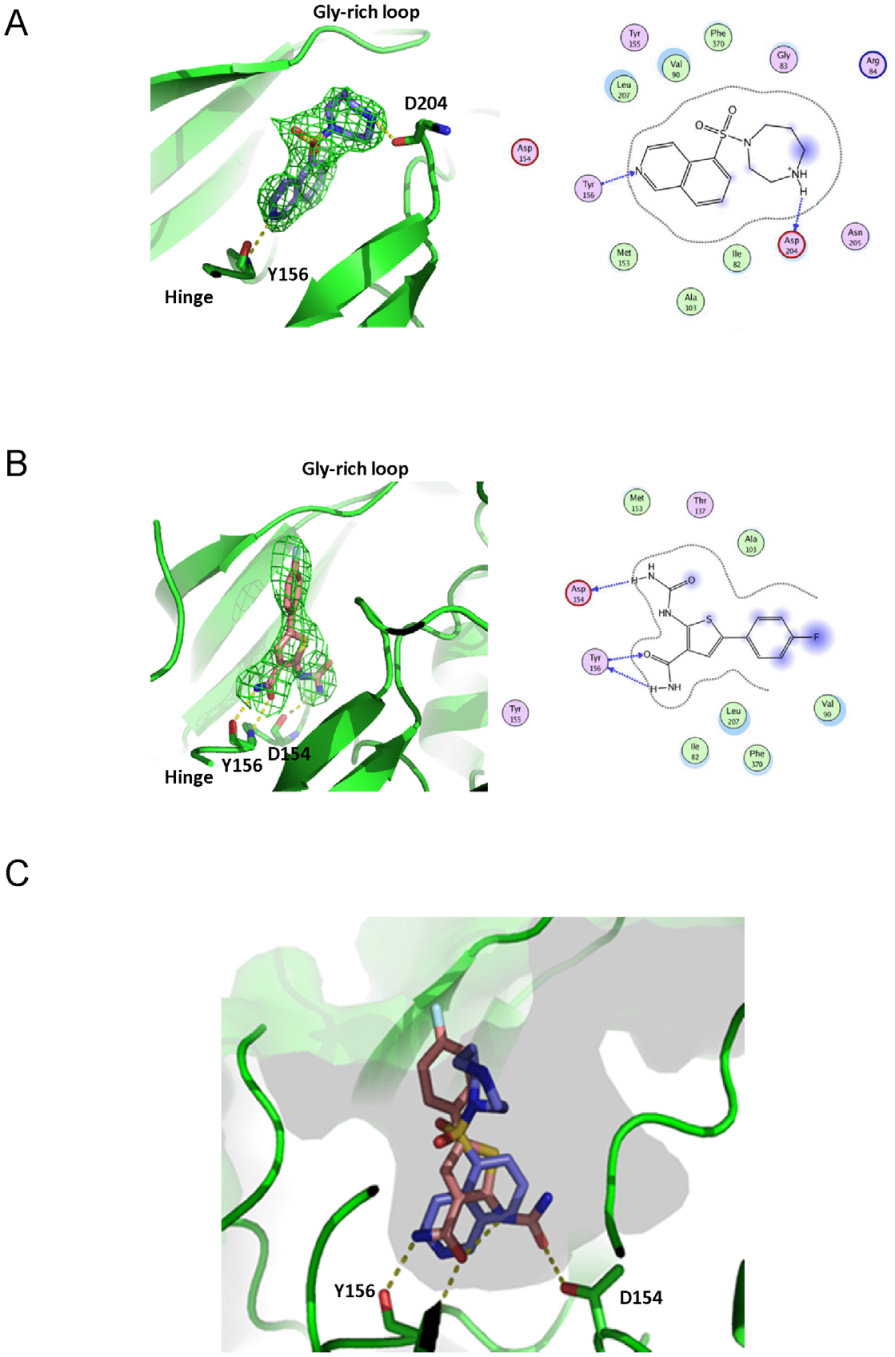
Positions of Fasudil and TPCA-1 in MRCKβ active site. (**A**) Structure of Fasudil bound to the active site of MRCKβ. Hydrogen bonds are marked with yellow dashed lines. The Fo–Fc omit map is shown contoured at 2σ, with the inhibitor modeled in the density. In the topology diagram, surface contour is shown with a gray dashed line and hydrogen bonds with blue dashed lines. Hydrophobic residues lining the cavity are shown in light green circles, and polar residues with light magenta. Blue shading indicates solvent exposed ligand groups. (**B**) Structure of TPCA-1 bound to the active site of MRCKβ. (**C**) Overlay of the structures of Fasudil and TPCA-1 bound to the active site of MRCKβ.

Both of the compounds crystallized here, Fasudil and TPCA-1, bind to the hinge region of the active site of MRCKβ (Figure 5). Fasudil and its derivatives have been previously crystallized with a number of AGC kinases, including ROCK1 and ROCK2 [30], [31]. The binding mode observed with MRCKβ does indeed reflect those observed in previously determined structures. The isoquinoline moiety forms a hydrogen bond to the hinge backbone of residue Y156 (Figure 5A). The homopiperazine ring further enhances the binding to the active site by linking the backbone of D204 and side chain of N205. These contacts are effectively identical to those seen in the Fasudil-ROCK complexes, and this is also reflected in equivalent IC_50_ values that have been obtained for these enzymes. There are two additional Fasudil molecules visible in the asymmetric unit, stacked between symmetry-related protein molecules (Figure 3A). Both of the molecules form hydrogen bonds to residue E252 but this binding site is unlikely to exist in solution as the sides of the binding cavity stacking the compound do not belong to a biologically relevant protein complex. Consequently, the binding observed at this location is likely to be non-specific and an artifact of the crystallization process.

TPCA-1, an inhibitor of IKK-2 [26], has not been previously crystallized with a kinase domain. This molecule makes hinge hydrogen bonding interactions through the amide group to the main chain of Y156 (Figure 5B). Furthermore, the carbamoylamino-moiety makes an additional hydrogen bond to the main chain of D154, and could further contribute to binding affinity through water-mediated hydrogen bonds. The fluorophenyl group points out from the active site.

An overlay of the two compounds indicates that they occupy similar space within the hinge-binding region, with both the homopiperazine ring of Fasudil and fluorophenyl moiety of TPCA-1 pointing out from the active site groove in a similar direction (Figure 5C).

## Discussion

Previous studies have shown that the combination of MRCK as well as ROCK inhibition has greater effects in blocking the invasiveness of tumor cells than inhibition of either kinase alone [17]. Similarly, the combined requirement for ROCK and MRCK as regulators of actomyosin contractility has been identified in ephrinB2-Fc induced endothelial cell retraction [35] and during *C. elegans* embryonic elongation [36]. Interestingly, the combination of ROCK and MRCK was also identified as being important regulators of human keratinocyte proliferation, although the mechanism for these observations was not established in this study [37]. In addition, MRCK has been shown to independently contribute to tumor cell invasion by contributing to the formation of single-cell invasion tunnels (SCIT) in 3D collagen matrices produced by membrane-type-1 matrix metalloproteinase activity [38] and by allowing squamous cell carcinoma cells to follow SCITs made by cancer-associated fibroblasts [23]. These studies indicate that there a number of ways that MRCK, either alone or in combination with ROCK, contributes to cancer. Although there is information about increased MRCK expression in tumors [39], it may also be the case that MRCK activity rather than expression is altered in cancers. Similar to the activating mutations identified in ROCK1 [40], sequencing of cancer genomes revealed mutations in MRCKα and MRCKβ that would likely increase their specific activity. The activity of Rho family GTPases such as Cdc42 may be up-regulated in tumor cells via increased protein expression [41], [42] or by increased activation from extracellular signals in the tumor environment (such as growth factors). Future studies will likely identify additional situations in which enhanced MRCK activity contributes to cancer growth and progression.

These findings would make it seem logical that the best course of action would be to develop inhibitors that simultaneously inhibited MRCK and ROCK. However, ROCK inhibitors have been shown to have profound effects on blood pressure that could present dose-limiting adverse cardiovascular effects [43]. It has been suggested that these effects are mediated by ROCK1, therefore, ROCK2 selective inhibitors have been developed to circumvent the adverse effects associated with non-isoform specific ROCK inhibitors [44]–[46]. If it were possible to avoid hypotensive effects by making ROCK inhibitors that were selective for ROCK2 over ROCK1, then it might also be possible to make inhibitors that blocked both MRCK isoforms and ROCK2 with selectivity over ROCK1. Given that ROCK inhibitors such as Fasudil also bind to and inhibit MRCK (Figures 2, 3 5), making inhibitors that potently block MRCK and ROCK should be possible, although the additional selectivity over ROCK1 will be challenging. [30], [31]The structure of MRCKβ reported in this study will provide better understanding of differences between AGC kinases and facilitate structure based development of specific inhibitors.

### Comparison of MRCK to other relevant AGC kinases

**Figure 6:**
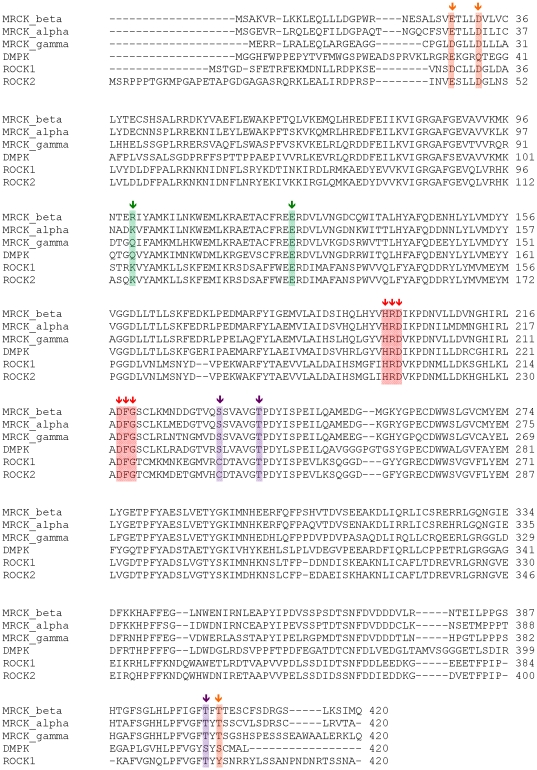
Sequence alignment of MRCKβ and the most closely related AGC kinases. Sequences have been truncated after the C-terminal lobe. The conserved HRD and DFG motifs have been highlighted with red arrows, and the conserved salt bridge at the active site with green arrows. Predicted phosphorylation sites have been indicated with magenta arrows, and the residues involved in hydrogen bonds between the C- and N-termini at the dimerization domain are indicated by orange arrows.

Development of MRCK-specific or MRCK/ROCK2-specific inhibitors relies on the structural differences between the different AGC kinases in question (Figure 6). MRCKα and MRCKβ share 62% identity over the full-length sequence and 77% identity across the kinase domain. The residues lining the nucleotide-binding site are fully conserved – therefore, selectivity for a specific isoform of MRCK is unlikely to be achievable by ATP-competitive inhibitors.

**Figure 7:**
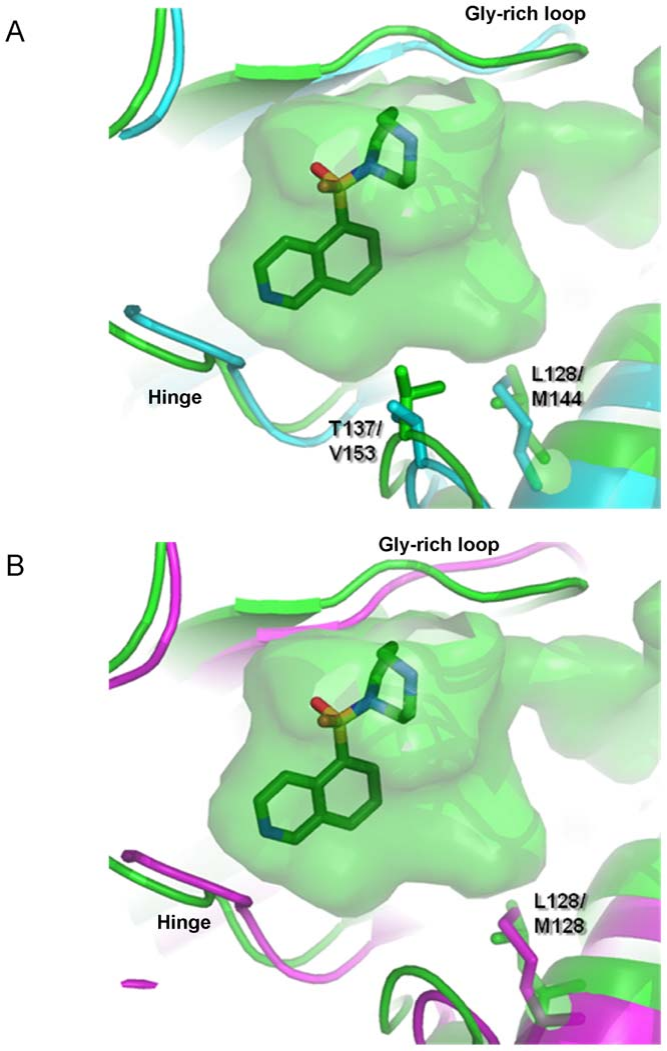
An overlay of MRCKβ (green) with ROCK2 (A; cyan) and ROCK1 (B; magenta) highlights the very subtle structural differences that could be exploited in developing specific inhibitors. The surface shown is the nucleotide binding cavity of MRCKβ, with Fasudil bound at the ATP binding site. Apart from the highlighted residues, the ATP binding sites of the three kinases are identical at sequence level.

Selectivity over ROCK isoforms, on the other hand, would seem more feasible considering the differences at the structural level. ROCK2 and MRCKβ are approximately 46% and 75% identical in the kinase domain and ATP site, respectively. ROCK2 has a proline in the hinge region, although this doesn't seem to have a major impact on the structure. Key substitutions between ROCK2 and MRCKβ are V153 (ROCK2)/T137 (MRCKβ) and M144 (ROCK2)/L128 (MRCKβ). These differences could be used to design selective inhibitors for MRCKβ (Figure 7A). ROCK1 and MRCKβ share approximately 47% identity across the kinase domain and 93% in the ATP site. There are only two substitutions in the active site, one of which points out of the site. The other is L128 (MRCKβ)/M128 (ROCK1), resulting only in a very subtle difference in surface topology (Figure 7B). Consequently, achieving selectivity against ROCK1 might be challenging and possibly requires extending compounds out from the ATP-binding site.

To conclude, the results shown in this study indicate that development of highly potent and specific inhibitors of these AGC kinases could be challenging, but the methods now available for structural studies of both MRCK and ROCK kinases should allow iterative drug development approaches.

## Materials and Methods

### Cell culture

MDA-MB-231-luciferase (Caliper LifeScience) were grown in DMEM with 10% fetal bovine serum (FBS), 2 mM L-glutamine plus 10 Units/ml penicillin and 10 µg/ml streptomycin at 37°C in 5% CO_2_ in a humidified incubator.

### siRNA Transfection

All siRNA reagents were obtained from Dharmacon RNA Technologies. All siRNA sequences available on request. Oligofectamine was used for transfection of siRNA on sub-confluent cells, according to manufacturer's instructions.

### Cell extraction and immunoblot analysis

RIPA lysates were prepared as described previously [47]. Whole cell lysates (60 µg) were separated by SDS-PAGE, transferred to Protran nitrocellulose membranes (Whatman), probed with primary antibodies and appropriate HRP-conjugated secondary antibodies (Pierce). Blots were visualized using ECL (Pierce) or Supersignal West Femto (Pierce) according to manufacturer's instructions. Antibodies were from: MRCKα (611584) was from BD Transduction Laboratories; MRCKβ (H00009578-A01) was from Abnova; MRCKα/β (MANDM1 6G8) from Glenn Morris (Centre for Inherited Neuromuscular Disease, Oswestry UK) [48], [49]; ROCK1 (61136) and ROCK2 (610623) were from BD Transduction Laboratories.

### Inverse invasion assays

Cell invasion assays were as previously described [25]. Complete Matrigel was thawed on ice, diluted 1∶1 with PBS and then 100 µl of diluted matrigel pipetted into Transwell inserts in a 24 well tissue culture plate and left to incubate for 30 minutes at 37°C to set. During this time cell suspensions of 3×10^5^ cells per ml from each condition were prepared in their normal growth medium. When the Matrigel had set, the Transwell inserts were inverted and 100 µl of cell suspension pipetted onto the filter. Transwell inserts were then carefully covered with the base of the 24 well tissue culture plate, making contact with each cell suspension droplet, and the plate then incubated in the inverted state for 4 hours to allow cell attachment. Following this time, plates were turned right-side-up and each Transwell insert washed with 3×1 ml serum-free DMEM and finally placed in a well in containing any additional inhibitors as indicated. DMEM plus 10% FBS was gently pipetted on top of the set Matrigel/PBS mixture and incubated for 5 days at 37°C. Propoidium iodide (PI) was used to visualize nuclei following fixation with paraformaldehyde, cells were imaged by confocal microscopy with a 20× objective with optical Z-sections scanned at 15 µm intervals moving up from the underside of the filter into the matrigel. ImageJ software was used for quantification. Statistical analysis was performed by ANOVA followed by Bonferroni's post-hoc test of significance, with p>0.05 selected as the cut-off.

### Cloning and protein expression

The cDNA encoding the human MRCKβ kinase domain (residues 2–417) with an N-terminal (His)_6_ tag and TEV protease cleavage site was codon optimized for expression in *E. coli*, synthesized and cloned into the Nde I and EcoR I restriction sites of pET21a (Novagen) by Blue Heron. MRCKβ 2–417 was expressed in *E. coli* BL21 (DE3). Transformed cells were grown in Terrific Broth at 30°C until A_600_ nm reached 0.6–0.9, and induced with 1 mM IPTG, followed by incubation at 18°C for 12–15 hrs. Pelleted cells were resuspended in lysis buffer (50 mM Hepes pH 7.5, 150 mM NaCl, 0.5 mM MgCl_2_, 20 mM imidazole, 0.05% mercaptoethanol, 1% phosphatase inhibitor cocktails 1 & 2 [Sigma], Protease inhibitor cocktail [Roche EDTA-free]) and lysed by sonication. Following centrifugal clarification (48,000×*g* for 30 min), supernatant was incubated with Ni-NTA Sepharose 6 FF beads (GE Healthcare) for 2.5 hr at 4°C. Weaker binding components were sequentially removed with 20 mM imidazole, 40 mM imidazole or 80 mM imidazole in ‘wash buffer’ (50 mM Hepes pH 7.5, 500 mM NaCl, 0.5 mM MgCl_2_, 0.05% mercaptoethanol). Beads were packed into a XK16/60 column (GE Healthcare) before bound protein was step eluted with 500 mM imidazole in ‘Wash Buffer’. Fractions were analyzed by UV, pooled and purified further by size exclusion chromatography (HiLoad 26/60 Superdex) using crystallization buffer (25 mM Hepes pH 7.5, 150 mM NaCl, 1 mM MgCl_2_, 1 mM DTT). Fractions containing dimeric MRCKβ were pooled, concentrated (Vivaspin 6, 10K cutoff), flash frozen and stored at −80°C.

### Crystallization and data collection

Crystals of MRCKβ 2–417 were obtained by incubating the protein (∼15 mg/mL concentration) with 1 mM of inhibitors (prepared from 50 mM DMSO stocks) overnight and setting up commercial crystallization screens the following morning. Plate-like crystals were observed after the protein was incubated at 4°C in hanging drops over well solution consisting of 15–25% PEG3350, 0.1 M BisTris pH 5.6, 0.2 M Ammonium Sulfate and 0.1 M Potassium/Sodium Tartrate. Crystals were frozen in well solution supplemented with 20% ethylene glycol. Data was collected at Diamond and Soleil.

### Data processing and structure determination

Data were processed using Mosflm [50]. The data collection statistics are presented in Table 3. The structure of MRCK β was solved using the structure of DMPK as the search model for molecular replacement using Phaser [51]. Refinement was carried out using Refmac [52] and the structures were built by using COOT [53].

**Table 3:** Data collection and refinement statistics.

Data set	MRCKβ-Fasudil	MRCKβ-TPCA-1
Synchrotron source	DIAMOND I03	Soleil PROXIMA1
Detector	Quantum-315r	Quantum-315r
Wavelength (Å)	0.98	0.98
Resolution (Å)	40.0-2.15 (2.27-2.15)	40.0-2.65 (2.79-2.65)
Rmerge (%)	11.6 (46.7)	11.1 (47.2)
Total observations	212536 (21206)	82188(10607)
Total unique observations	47854 (6046)	24954(3450)
Mean ((I/sd(I))	7.9 (2.4)	7.4 (2.4)
Completeness (%)	97.1 (84.3)	94.7 (89.8)
Multiplicity	4.4 (3.5)	3.3 (3.1)
Cell parameters (a,b,c (Å); β (°))	44.94 123.61, 84.78; 101.2	44.70, 123.45, 85.31; 100.6
Spacegroup	P 2_1_	P 2_1_
Molecules/AU	2	2
R factor (%)	21.0	20.5
R_free_ (%)	26.9	28.6
PDB ID	3TKU	3QFV

Values in parentheses represent data in the highest resolution shell.

### Enzyme assays

Activity assays for MRCKα and β were performed using an IMAP fluorescence polarization assay kit (Molecular Devices Inc.). 8–12 nM MRCKα or MRCKβ (Millipore) was incubated for 60 minutes at room temperature with 100 nM FAM-S6-ribosomal protein derived peptide (Molecular Devices Inc.) in the presence of 0.7 µM ATP and 0.5 mM Mg^2+^ in 20 mM Tris buffer (pH 7.4) containing 0.01% Tween-20 and 1 mM DTT. Dose response inhibition analyses were performed over a drug concentration range from 0.002–40 µM, and single point inhibition was assessed at 3 or 30 µM drug. Kinase reactions were stopped with 2 assay volumes of 0.25% (v/v) IMAP binding reagent in 1× IMAP binding buffer A (Molecular Devices Inc.). After two hours incubation to allow binding reagent to bind phosphorylated peptide, fluorescence polarization was measured on a PHERAstar plate reader (BMG Labtech GmbH) at excitation and emission wavelengths of 485 nm and 520 nm respectively. Percent inhibition was calculated using no inhibitor and no enzyme controls for 0 and 100% inhibition, respectively.
